# Clinical predictors of lacunar syndrome not due to lacunar infarction

**DOI:** 10.1186/1471-2377-10-31

**Published:** 2010-05-18

**Authors:** Adrià Arboix, Joan Massons, Luis García-Eroles, Cecilia Targa, Emili Comes, Olga Parra

**Affiliations:** 1 Unit of Cerebrovascular Diseases, Service of Neurology, Hospital Universitari del Sagrat Cor, Universitat de Barcelona, Barcelona, Spain; 2 Unit of Organization, Planning and Information Systems, Consorci Sanitari del Maresme, Barcelona, Spain; 3 Service of Pneumology, Hospital Universitat de Barcelona, Barcelona, Spain; 4 CIBER de Enfermedades Respiratorias (CB06/06), Instituto Carlos III, Madrid, Spain

## Abstract

**Background:**

Lacunar syndrome not due to lacunar infarct is poorly characterised. This single centre, retrospective study was conducted to describe the clinical characteristics of patients with lacunar syndrome not due to lacunar infarct and to identify clinical predictors of this variant of lacunar stroke.

**Methods**

A total of 146 patients with lacunar syndrome not due to lacunar infarction were included in the "Sagrat Cor Hospital of Barcelona Stroke Registry" during a period of 19 years (1986-2004). Data from stroke patients are entered in the stroke registry following a standardized protocol with 161 items regarding demographics, risk factors, clinical features, laboratory and neuroimaging data, complications and outcome. The characteristics of these 146 patients with lacunar syndrome not due to lacunar infarct were compared with those of the 733 patients with lacunar infarction.

**Results**

Lacunar syndrome not due to lacunar infarct accounted for 16.6% (146/879) of all cases of lacunar stroke. Subtypes of lacunar syndromes included pure motor stroke in 63 patients, sensorimotor stroke in 51, pure sensory stroke in 14, atypical lacunar syndrome in 9, ataxic hemiparesis in 5 and dysarthria-clumsy hand in 4. Valvular heart disease, atrial fibrillation, sudden onset, limb weakness and sensory symptoms were significantly more frequent among patients with lacunar syndrome not due to lacunar infarct than in those with lacunar infarction, whereas diabetes was less frequent. In the multivariate analysis, atrial fibrillation (OR = 4.62), sensorimotor stroke (OR = 4.05), limb weakness (OR = 2.09), sudden onset (OR = 2.06) and age (OR = 0.96) were independent predictors of lacunar syndrome not due to lacunar infarct.

**Conclusions**

Although lacunar syndromes are highly suggestive of small deep cerebral infarctions, lacunar syndromes not due to lacunar infarcts are found in 16.6% of cases. The presence of sensorimotor stroke, limb weakness and sudden onset in a patient with atrial fibrillation should alert the clinician to the possibility of a lacunar syndrome not due to a lacunar infarct.

## Background

Lacunar syndromes are usually caused by lacunar infarctions [1,2]. However, other stroke subtypes, including small intracerebral haemorrhages, spontaneous subdural haematomas or non-lacunar cerebral infarctions may occasionally be the aetiology of lacunar syndromes. Lacunar syndromes not due to lacunar infarcts are poorly defined [3-5]. Therefore, the aim of this study was to describe the clinical characteristics of patients with lacunar syndrome not due to lacunar infarct and to identify clinical predictors of this variant of lacunar stroke. As far as we are aware, the present series of patients with lacunar syndrome not due to lacunar infarct collected from a prospective stroke registry represents the largest experience of this infrequent stroke subtype reported in the literature.

## Methods

The database of the "Sagrat Cor Hospital of Barcelona Stroke Registry" with data of 3808 acute stroke patients was searched for those with a diagnosis of first-ever lacunar stroke who were admitted consecutively to the Department of Neurology of the Sagrat Cor Hospital (an acute-care 350-bed teaching hospital in the city of Barcelona) between January 1986 and December 2004. Details of this on-going hospital-based stroke registry have been previously reported [6]. Data from stroke patients are entered following a standardised protocol with 161 items regarding demographics, risk factors, clinical features, laboratory and neuroimaging data, complications and outcome. Subtypes of stroke were classified according to the Cerebrovascular Study Group of the Spanish Society of Neurology, which is similar to the National Institute of Neurological Disorders and Stroke classification [7].

For the purpose of this study, all cases of first-ever lacunar stroke diagnosed in 879 patients were collected. Lacunar infarcts were defined [6,8] as (*a*) sudden or gradual onset of a focal neurological deficit lasting > 24 hours of the type described in the common lacunar syndromes (pure motor hemiparesis, pure sensory stroke, sensorimotor stroke, ataxic hemiparesis, dysarthria-clumsy hand and atypical lacunar syndromes); (*b*) brain CT scans or MRI were either normal or demonstrated only small, localised brain lesions with diameter smaller than 20 mm that seemed appropriate for the neurological deficits, and (*c*) absence of cortical ischaemia, cervical carotid stenosis (> 50% diameter) or major source for cardioembolic stroke. Lacunar syndromes not due to lacunar infarcts were defined as sudden or gradual onset of a focal neurological deficit lasting > 24 hours of the type described in the common lacunar syndrome (pure motor hemiparesis, pure sensory stroke, sensorimotor stroke, ataxic hemiparesis, dysarthria-clumsy hand and atypical lacunar syndrome[8]) secondary to non-lacunar ischaemic stroke (cortical or subcortical lesions > 20 mm with atherothrombotic, cardioembolic, unusual etiology or unknown etiology) or haemorrhagic stroke (intracerebral haemorrhage or spontaneous subdural haematoma).

All patients were admitted to the hospital within 48 hours of the onset of symptoms. Systematic investigations included clinical examination, 12-lead electrocardiogram, chest radiology, and standard blood tests including serum cholesterol concentration and lipid profile. A brain CT scan and/or MRI (41%) was performed within this first week of hospital admission. Angio-MRI was performed in 38% of the patients. Other investigations included Doppler ultrasonography of the supara-aortic trunks in 65.5% of the cases, arterial digital subtraction angiography in 8%, 2-dimensional echocardiography in 41.2%, and lumbar puncture in 2.8%. Demographic data, vascular risk factors, clinical features, neuroimaging findings, and outcome were recorded. Details of variables prospectively registered in the database have been previously reported [6]. Medical complications (respiratory, urinary, cardiac, vascular and infectious) and mortality during the acute phase of the disease was assessed. Causes of death were analyzed according to criteria of Silver et al. [9].

### Statistical analysis

Demographic characteristics, risk factors, aetiology of stroke, subtypes of stroke and of lacunar syndromes, clinical events and outcome of patients with lacunar syndrome not due to lacunar infarct and those with lacunar infarction were compared with the analysis of variance (ANOVA) (with Bonferroni's correction when necessary) and the chi-square (χ^2^) test (with Yate's correction when necessary). Variables were subjected to multivariate analysis with a logistic regression procedure and forward stepwise selection if *P *< 0.10 after univariate testing. The effect of variables on the presence of a lacunar syndrome not due to lacunar infarct was analysed in a predictive model based on demographic, vascular risk factors and clinical, variables, in which lacunar syndrome not due to lacunar infarct was the dependent variable. Age was used as a continuous variable with a constant odds ratio for each year. The odds ratio (OR) and 95% confidence intervals (CI) were calculated from the beta coefficients and standard errors. Statistical significance was set at *P *< 0.05.

## Results

The diagnosis of lacunar syndrome not due to lacunar infarct was established in 146 (16.6%) of all 879 consecutive patients with first-ever lacunar stroke. There were 82 men and 64 women with a mean (SD) age of 71.6 (12.6) years. Twenty-six patients aged 85 years or older. The following vascular risk factors in a decreasing order of frequency were observed: hypertension (73.3%), atrial fibrillation (30.1%), diabetes mellitus (21.2%), hyperlipidemia (19.9%), ischaemic heart disease (15.8%) and heavy smoking (> 20 cigarettes/day) (13%). As shown in Table 1, subtypes of lacunar syndromes not due to lacunar stroke included pure motor stroke in 43% (63/146) of patients, sensorimotor stroke in 35% (52/146), pure sensory stroke in 9.5% (14/146), atypical lacunar syndrome in 6.2% (9/146), ataxic hemiparesis in 3.5% (5/146) and dysarthria-clumsy hand in 2.7% (4/146). In relation to the total number of patients with the different subtypes of lacunar syndromes, those not due to lacunar stroke accounted for 15.2% (63/415) of pure motor strokes, 38.8% (52/143) of sensorimotor strokes, 9.9% (14/141) of pure sensory strokes, 9.3% (9/27) of atypical lacunar syndromes, 17.2% (5/29) of ataxic hemiparesis and 6.3% (4/63) of dysarthria-clumsy hand.

**Table 1:** Results of univariate analysis in patients with lacunar syndrome not due to lacunar infarction compared with patients with lacunar infarct


**Data**	**Lacunar syndrome not due to lacunar infarct**	**Lacunar infarct**	***P *value**

Total patients	146	733	
Males	82 (56.2)	423 (57.7)	0.730
Age, years, mean (SD)	72.9 (12.6)	74.1 (10.2)	0.285
Age ≥ 85 years	26 (17.8)	110 (15.0)	0.393
Cardiovascular risk factors			
Hypertension	107 (73.3)	525 (71.6)	0.683
Diabetes mellitus	31 (21.2)	218 (29.7)	0.037
Valvular heart disease	10 (6.8)	21 (2.9)	0.017
Ischaemic heart disease	23 (15.8)	104 (14.2)	0.623
Atrial fibrillation	44 (30.1)	81 (11.1)	0.0001
Congestive heart failure	4 (2.7)	24 (3.3)	0.737
Transient ischaemic attack	12 (8.2)	80 (10.9)	0.331
Previous cerebral infarction	16 (11)	117 (16)	0.123
Periphery arterial disease	17 (11.6)	57 (7.8)	0.124
Obesity	8 (5.5)	47 (6.4)	0.671
Alcohol abuse (> 80 g/day)	7 (4.8)	21 (2.9)	0.340
Heavy smoking (> 20 cigarettes/day)	19 (13)	86 (11.7)	0.663
Hyperlipidemia	29 (19.9)	166 (22.6)	0.460
Clinical features			
Sudden onset (minutes)	78 (53.4)	310 (42.3)	0.013
Headache	15 (0.3)	68 (9.3)	0.707
Early seizures	1 (0.7)	0	0.369
Limb weakness	129 (88.4)	554 (75.6)	0.001
Sensory symptoms	64 (43.8)	231 (31.5)	0.004
Speech disturbances	52 (35.6)	311 (42.4)	0.127
Ataxia	8 (5.5)	50 (6.8)	0.551
Cranial nerve palsy	4 (2.7)	21 (2.9)	1
Lacunar syndromes			0.0001
Pure motor hemiparesis	63 (43)	352 (48)	
Pure sensory stroke	14 (9.5)	127 (17.3)	
Sensorimotor stroke	51 (35)	83 (11.3)	
Dysarthria-clumsy hand	4 (2.7)	59 (8)	
Ataxic hemiparesis	5 (3.5)	24 (3.3)	
Atypical lacunar syndrome*	9 (6.2)	88 (12)	
Outcome			
Symptom-free at discharge	27 (18.5)	166 (22.6)	0.268
Respiratory events	5 (3.4)	17 (2.3)	0.624
Urinary infection	7 (4.8)	20 (2.7)	0.290
Cardiac events	5 (3.4)	8 (1.1)	0.079
Vascular complications	1 (0.7)	4 (0.5)	1
Infectious complications	7 (4.8)	28 (3.8)	0.582
Length of hospital stay, days, (median, interquartile range [25th-75th percentile)	12 (8.5-20)	10 (7-14)	0.001
In-hospital deaths	1 (0.7)	4 (0.5)	1


Data are *n *(%) unless otherwise stated.

*Dysarthria facial paresis in 3 patients, isolated dysarthria in 2, bilateral pure motor hemiparesis with transient subcortical aphasia in 2, pure motor hemiparesis with transient internuclear ophthalmoplegia in 1, and paramedian thalamic infarct syndrome in 1.

In relation to the aetiology of the different stroke subtypes included in the database, lacunar syndrome not due to lacunar infarct occurred in 10.5% (4/38) of cases of spontaneous subdural haematoma, 9.1% (37/408) of primary intracerebral haemorrhage, 7% (54/770) of atherothrombotic infarction, 5% (38/763) of cardioembolic infarction, 3.4% (11/324) of infarcts of unknown cause and 1.8% (2/114) of infarcts of unusual aetiology.

One patient died (in-hospital mortality rate 0.7%). This patient died suddenly of an unknown cause. The median length of hospitalization was 12 days (interquartile range 8.5-20 days). Only 27 (18.5%) patients were symptom-free at the time of hospital discharge.

As shown in Table 1, valvular heart disease, atrial fibrillation, sudden onset, limb weakness and sensory symptoms were significantly more frequent among patients with lacunar syndrome not due to lacunar infarct than in those with lacunar infarction, whereas diabetes was less frequent. In the multivariate analysis, atrial fibrillation (OR = 4.62), sensorimotor stroke (OR = 4.05), limb weakness (OR = 2.09), sudden onset (OR = 2.06) and age (OR = 0.96) were independent predictors of lacunar syndrome not due to lacunar infarct (Table 2).

**Table 2:** Variables associated with lacunar syndrome not due to lacunar infarct


**Variable**	**β**	**SE (β)**	**Odds ratio (95% CI)**	***p***

Model based on demographics, vascular risk factors and clinical variables*				
Atrial fibrillation	1.532	0.301	4.62 (2.56-8.36)	0.0001
Sensorimotor stroke	1.396	0.294	4.05 (2.28-7.19)	0.0001
Limb weakness	0.738	0.363	2.09 (1.03-4.26)	0.042
Sudden onset	0.721	0.253	2.06 (1.25-3.37)	0.004
Age	-0.038	0.011	0.96 (0.94-0.98)	0.001


* β = -0.521; SE (β) = 0.825; goodness-of-fit × ^2 ^= 3.675; *df *= 8; *P *= 0.885; area under the ROC curve = 0.753; sensitivity 67%; specificity 74%; positive predictive value 33%; negative predictive value 92%; correct classification 73%.

## Discussion

In the present series, the incidence of lacunar syndrome not due to lacunar infarct was 16.6%. In earlier studies, the frequency of acute lacunar syndromes varied between 6% and 42% [3,14,15] but these series studied patients with pure motor stroke only [10] or included patients with non-vascular aetiologies (multiple sclerosis, cerebral abscess, glioblastoma) [3,10]. In small series of patients, studies in which MRI examination was performed during the acute phase of stroke, it was observed that lacunar syndromes were predictors of lacunar infarcts [11-13]. In one of these studies [12], cortical involvement was found in only 2 out of 23 patients. In another study [13], acute diffusion-weighted MRI altered the final diagnosis of small vessel occlusion in 40% of 73 patients with a lacunar syndrome [13]. Most of these 'false positive' cases are attributable to large artery or cardiogenic embolic stroke. Therefore, the present results based on a clinical series of 146 consecutive patients diagnosed of lacunar syndrome not due to lacunar infarct, --the largest reported in the literature up to the present time--, reinforce the lacunar hypothesis, which states that in the presence of a lacunar syndrome defined only by clinical data, lacunar infarction is the most common aetiology [4].

In relation to aetiological stroke subtypes, lacunar syndrome not due to lacunar infarct was found in 10.5% of cases of spontaneous subdural haematoma and in 9.1% of primary intracerebral haemorrhage. Subdural haematomas and intracerebral haemorrhage have been reported to cause non-ischaemic lacunar syndromes not due to lacunar infarct [3,14]. Likewise, lacunar syndromes not due to lacunar infarcts were documented in 7% of atherothrombotic infarction, 5% of cardioembolic infarction, 3.4% of infarcts of unknown cause and 1.8% of infarcts of unusual aetiology, which is in agreement with other studies showing that lacunar syndromes may be caused by extensive non-lacunar infarctions, mainly of subcortical topography and less frequently cortical infracts [3,13].

A sensorimotor stroke is possible from a single lacune and constitutes one of the five classic or typical lacunar syndromes [15-17]. However, there has been some disagreement whether sensorimotor stroke should be included among the lacunar syndromes, mainly because of limited pathological confirmation [16]. In our study, sensorimotor stroke may not be caused by a lacunar infarct in 38.8% of cases. In addition, sensorimotor stroke was an independent predictor of lacunar syndrome mot caused by lacunar infarct in the logistic regression model. These results are consistent with the study of Staaf et al. [18] in which 31% of patients with sensorimotor stroke had a potential cardioembolic source, large artery disease or infarcts not compatible with perforating artery disease. Therefore, in about one in three patients with sensorimotor stroke, aetiologies other than ischaemic small vessel disease may be present.

Atrial fibrillation was also independently associated with lacunar syndrome not due to lacunar infarct. This finding agrees with other studies showing that atrial fibrillation is less frequent in lacunar infarcts than in other non-lacunar strokes [5,19] and that patients with a lacunar syndrome but a territorial infarct more often had a cardioembolic source [5]. Cardioembolism constitutes a rare cause for acute lacunar infarcts [3]. Emboligenous cardiopathy as the only demonstrable cause has been found in 2.6-5% of lacunar infarctions [20,21]and its role in the aetiology of lacunar infarction is very rare. Macroembolism could be shown in one case originally described by Fisher and Curry related to pure motor hemiplegia [22]. In one series, it was noted that embolic cardiopathy as single aetiology was only seen in 4% of lacunar infarcts [23].

Limb weakness was more frequent in lacunar syndrome not due to lacunar infarct than in lacunar infarction because non-lacunar infarcts or intracerebral haemorrhages are likely to be more extensive that lacunes [3]. Sudden stroke onset was also more common in patients with lacunar syndrome not due to lacunar infarct. Patients with lacunar infarcts usually present a gradual onset of a focal neurological deficit [24,25], which is in contrast to cardioembolic stroke and also some cases of intraparenchymatous haemorrhages [26] characterised by sudden onset (minutes) of symptoms and maximal neurological deficit at onset. Patients with lacunar syndrome not due to lacunar infarct were younger than those with lacunar infarction, which agrees with other series previously reported [3,27].

It should be noted that a differentiation between patients with single infarcts and patients with multiple infarcts was not performed, nor the presence of coexistent white matter lesions was assessed. In these cases, MRI is the most adequate neuroimaging technique but only 41% of patients were studied by MRI within this first week of hospital admission. Limitations of the present findings include the single-center characteristics of the study and the fact that this was not a population-based study, as a result of which care should be taken to make inferences to other target populations beyond the subjects in the study.

## Conclusions

Although lacunar syndromes are highly suggestive of small deep cerebral infarctions, lacunar syndromes not due to lacunar infarcts are found in 16.6% of cases. The presence of sensorimotor stroke, limb weakness and sudden onset in a patient with atrial fibrillation should alert the clinician to the possibility of a lacunar syndrome not due to a lacunar infarct.

## List of abbreviations

CT: Computed tomography; MRI: Magnetic resonance imaging; OR: Odds ratio; SD: standard deviation.

## Competing interests

The authors declare that they have no competing interests.

## Authors' contributions

AA was the principal investigator, designed the study, diagnosed and took care of thepatients, contributed to analyze the data, interpreted the results, wrote the paper, andprepared the final draft. He was alsoresponsible for editorial decisions includingthe selection of the journal.

LGE was the statistician, participated in the study design, analysis andinterpretation of data, wrote the part of thepaper related to the statistical analysis, andapproved the final draft.

JM, CT, EC and OP participated in the collectionof data, medical care of the patients, analysis of results, review of the manuscript for intellectual content, and approved the final draft.

## Pre-publication history

The pre-publication history for this paper can be accessed here:

http://www.biomedcentral.com/1471-2377/10/31/prepub
